# A molecularly defined D1 medium spiny neuron subtype negatively regulates cocaine addiction

**DOI:** 10.1126/sciadv.abn3552

**Published:** 2022-08-12

**Authors:** Zheng-dong Zhao, Xiao Han, Renchao Chen, Yiqiong Liu, Aritra Bhattacherjee, Wenqiang Chen, Yi Zhang

**Affiliations:** ^1^ Howard Hughes Medical Institute, Boston Children’s Hospital, Boston, MA 02115, USA.; ^2^ Program in Cellular and Molecular Medicine, Boston Children’s Hospital, Boston, MA 02115, USA.; ^3^ Division of Hematology/Oncology, Department of Pediatrics, Boston Children’s Hospital, Boston, MA 02115, USA.; ^4^ Department of Genetics, Harvard Medical School, Boston, MA 02115, USA.; ^5^ Harvard Stem Cell Institute, WAB-149G, 200 Longwood Avenue, Boston, MA 02115, USA.

## Abstract

The striatum plays a critical role in regulating addiction-related behaviors. The conventional dichotomy model suggests that striatal D1/D2 medium spiny neurons (MSNs) positively/negatively regulate addiction-related behaviors. However, this model does not account for the neuronal heterogeneity and functional diversity of the striatum, and whether MSN subtypes beyond the pan-D1/D2 populations play distinct roles in drug addiction remains unknown. We characterized the role of a *tachykinin 2*–expressing D1 MSN subtype (*Tac2^+^*), present in both rodent and primate striatum, using cocaine addiction mouse models. We found that acute cocaine administration reduces *Tac2* neuronal activity, and cocaine conditioning alters neuronal response related to cocaine reward contextual associations. In addition, activation/inhibition of *Tac2^+^* neurons attenuates/promotes cocaine-induced conditioned place preference and cocaine intravenous self-administration. Furthermore, stimulation of the NAc-to-lateral hypothalamic projection of *Tac2^+^* neurons suppresses cocaine reward behavior. Our study reveals an unconventional negative regulatory function of D1 MSNs in drug addiction that operates in a subtype- and projection-specific manner.

## Teaser

We identified an unconventional role of a molecularly defined D1 MSN subtype in suppressing cocaine reward and addiction.

## INTRODUCTION

Drug addiction (or substance use disorder) is a chronic, relapsing disorder that features compulsive drug seeking and taking, causing incapability of self-control and harmful consequences (*1*). As an important component of the basal ganglia and brain reward circuitry, the nucleus accumbens (NAc) plays a critical role in processing reward and motivation-related information (*2*–*7*), and its role in drug addiction has been intensively studied for decades (*8*–*12*). Although these studies have provided important insights into the roles of NAc in regulating different aspects of addiction-related behavior, some important questions remain unanswered. For example, it has been shown that different subregions (such as “core” and “shell”) (*13*, *14*) and different inputs/outputs (*15*–*17*) of the NAc are involved in different aspects of addiction-related behavior. However, it is unknown whether the functional diversity of NAc can be attributed to its different neuronal substrates, and it remains challenging to explain some discordant findings regarding the causal roles of D1 medium spiny neurons (MSNs) in regulating reward-related behaviors (*10*, *17*, *18*). This is at least partially due to the incomplete understanding of the neuronal composition within the NAc.

The conventional direct/indirect pathway model, which divides the striatal projection neurons into D1 and D2 MSN, has inspired the understanding of the cellular, circuitry, and functional organization of the striatum since it was proposed (*19*–*24*). However, this model largely ignored the intrastriatal heterogeneity and thus cannot explain the substantial molecular, anatomic, and functional heterogeneity observed in the NAc (*18*, *25*–*28*). Consistent with this notion, recent single-cell RNA sequencing (scRNA-seq) and MERFISH (Multiplexed Error-Robust Fluorescence in situ Hybridization) studies have revealed rich neuron subtypes beyond the D1/D2 MSN dichotomy model (*29*–*31*), suggesting that the molecularly defined D1/D2 MSN subtypes may play different roles during the addictive process. Furthermore, some recent studies have revealed that functionally distinct subpopulations of striatal D1/D2 MSNs are involved in reinforcement learning and drug-related behavior (*18*, *28*, *32*–*38*). However, direct evidence of molecularly defined MSN subtypes (beyond pan-D1/D2 population) in regulating addiction-related behavior is still lacking.

To fill in this gap, we interrogated the function of *tachykinin 2* (*Tac2*)–expressing MSN, one of the 30 D1 MSN subtypes located in the NAc dorsomedial shell (*31*), in mouse models of cocaine addiction. By combining single-cell Ca^2+^ imaging and cell type–specific activity manipulation, we uncovered an unexpected role of *Tac2*^+^ neurons in negatively regulating cocaine reward and contingent drug taking, without affecting the psychostimulatory effect of cocaine. Our findings highlight the importance of using the cell type–specific approach in understanding drug addiction.

## RESULTS

### Tac2-expressing D1 MSN activity is suppressed by cocaine administration

**Fig. 1.**
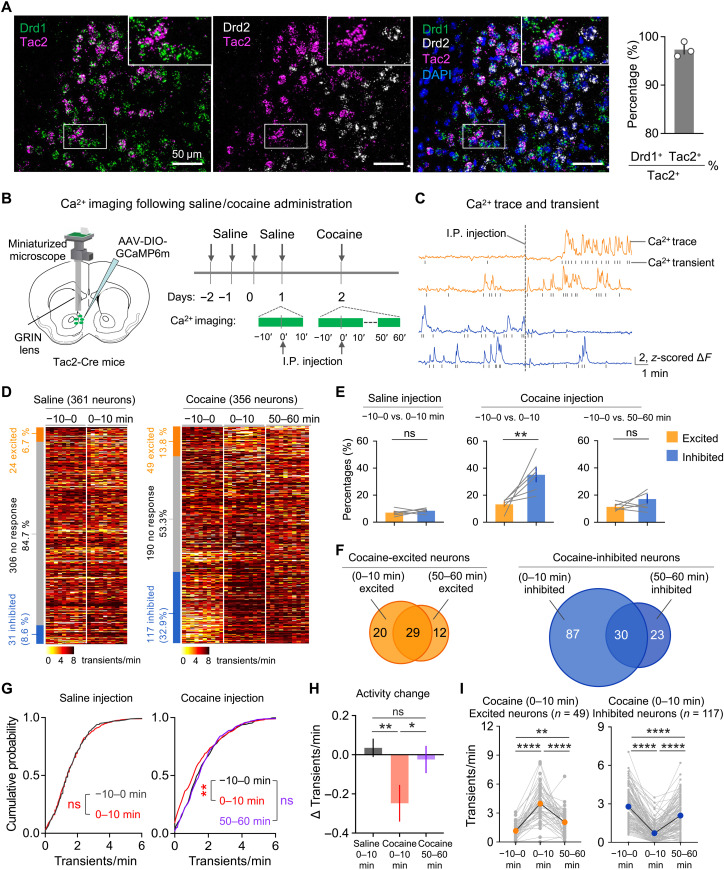
*Tac2*-expressing D1 MSNs in NAc respond to cocaine administration. (**A**) RNA FISH of *Drd1*, *Drd2*, and *Tac2*. High-magnification images of the boxed areas are on the top right. The *Drd1^+^ Tac2*^+^ double-positive cells account for 97.3 ± 1.5% of *Tac2*^+^ neurons (*n* = 3 mice; total, 548 cells; scale bars, 50 μm). (**B**) Diagram of calcium imaging following saline/cocaine administration. The recorded sessions including the preinjection phase (−10 to 0 min), postinjection initial phase (0 to 10 min), and decay phase (50 to 60 min) are indicated with green lines. (**C**) Representative calcium traces and transients of individual neurons that were activated (orange) or inhibited (blue) by cocaine injection. I.P., intraperitoneal. (**D**) Heatmap of calcium transient frequency of all neurons from six mice recorded following saline (left) or cocaine (right) injection. The percentage of neurons that were excited by (orange), inhibited by (blue), or had no response to (gray) treatment were shown in the stacked bar graph next to the heatmap. (**E**) Bar graphs showing the percentages of neurons that are excited (orange) or inhibited (blue) by saline (left) or cocaine (right) administration. (**F**) Venn diagram showing the overlap between the initial phase (0 to 10 min) and decay phase (50 to 60 min) of postinjection of cocaine-excited neurons (left) or cocaine-inhibited neurons (right). (**G**) Cumulative distribution of calcium transient frequencies around saline (left) and cocaine (right) injection (saline, *n* = 361; cocaine, *n* = 356). (**H**) Bar graph showing the calcium activity change before and after injection. (**I**) Average calcium transient frequency before and after cocaine injection from neurons that showed elevated (left, orange) or reduced (right, blue) activity during the initial phase (0 to 10 min) of cocaine injection. Data in (A), (E), (H), and (I) are presented as means ± SEM. The *P* values are calculated on the basis of statistical tests in table S1. *P ≤ 0.05; **P ≤ 0.01; ****P ≤ 0.0001; not significant (ns), *P* > 0.05.

scRNA-seq of mouse NAc neurons revealed that *Tac2* is selectively expressed in 1 of the 30 D1 MSN subtypes we recently identified that accounts for ~5% of the total NAc D1 MSNs (fig. S1A) (*31*). Similarly, a recent study in primate has identified an equivalent D1 archetype in the primate striatum (*39*). In situ RNA hybridization revealed that *Tac2*-expressing neurons are located in the dorsomedial part of the NAc shell and core (fig. S1B), a region associated with drug reward processing (*40*). Multicolor RNA hybridization further confirmed that *Tac2* is predominantly expressed in a subpopulation of D1 MSNs, with little overlap with D2 MSNs (Fig. 1A). The cellular and spatial properties of *Tac2*^+^ neurons suggest that they may be involved in drug reward–related behaviors.

To explore a potential role of *Tac2*^+^ neurons in drug-related behavior, we monitored *Tac2* neuronal activity using single-cell calcium imaging upon psychostimulant drug (cocaine) treatment. To this end, GCaMP6m, which reports calcium fluctuations and serves as a proxy for neuronal activity, was virally delivered into the NAc of *Tac2*-Cre mice (*41*), and the fluorescence signal was monitored by a skull-attached miniaturized microscope through a gradient-index (GRIN) lens implanted above the virus injection site (Fig. 1B and fig. S1, C and D). Since the cocaine levels in both brain and plasma peak within 10 min after administration, and decay 1 hour later (*42*, *43*), we performed calcium signal recording in one 10-min session during preinjection (baseline, −10 to 0 min) and two 10-min sessions (initial phase, 0 to 10 min; decay phase, 50 to 60 min) during postinjection (intraperitoneal injection) of cocaine (15 mg/kg) (Fig. 1B). After extracting calcium signal traces and corresponding calcium signal transients of individual neurons from raw imaging video (Fig. 1C and fig. S1E), we analyzed *Tac2* neuronal response to saline or cocaine administration. The calcium transient frequency from six mice (saline, 361 neurons; cocaine, 356 neurons) was sorted and presented as heatmaps (Fig. 1D). Upon saline injection, only a small proportion of *Tac2*^+^ neurons showed substantial changes in their activity (24 of 361 or 6.7% neuron increased activity, and 31 of 361 or 8.6% neuron decreased activity) (Fig. 1D). In contrast, cocaine injection significantly increased the number of responsive *Tac2*^+^ neurons, with 13.8% (49 of 356) and 32.9% (117 of 356) of recorded neurons showing increased and decreased activity, respectively, in the initial phase (0 to 10 min). The numbers of cocaine-responsive neurons declined in the decay phase (50 to 60 min), with 11.5% (41 of 356) and 14.9% (53 of 356) of recorded neurons showing increased and decreased activity, respectively (Fig. 1E). Among the cocaine initial phase–responsive neurons, 59.2% (29 of 49) of the cocaine-excited neurons and 25.6% (30 of 117) of the cocaine-inhibited neurons are respectively excited and inhibited in the decay phase (Fig. 1F), indicating that *Tac2*^+^ neurons exhibit dynamic response to cocaine administration. We observed that a 2.4-fold more *Tac2*^+^ neurons are inhibited by cocaine when compared to those excited by cocaine in the initial phase (Fig. 1E), and an overall decrease of *Tac2* neuronal activity following cocaine injection was observed, which was not the case in the saline treatment (Fig. 1, G to H). The neuronal activity of cocaine-excited/inhibited *Tac2*^+^ neurons in the initial phase was substantially altered following cocaine injection and later partially recovered in the decay phase (Fig. 1I). To further validate these findings, we analyzed calcium activity traces before and after saline or cocaine injection, and we identified 3.0-fold more cocaine-inhibited neurons than cocaine-excited neurons in the initial phase (fig. S1, F to H). Collectively, these results demonstrate that acute cocaine administration modulates the activity of a significant percentage of *Tac2*^+^ neurons, with the majority of them showing decreased neuronal activity.

### Cocaine place conditioning modulates the activity of Tac2^+^ neurons

**Fig. 2.**
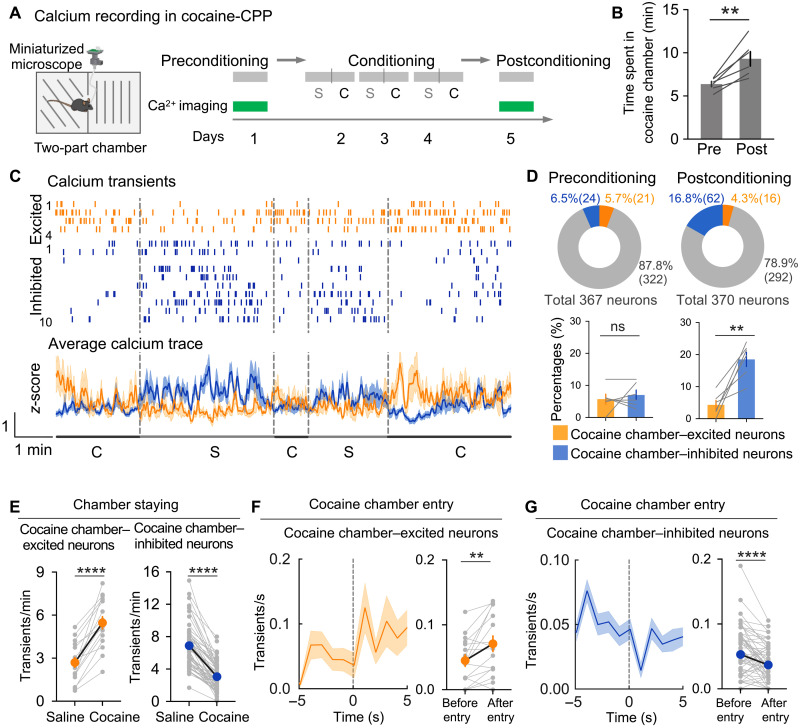
Cocaine place conditioning modulates *Tac2* neuronal activities that are associated with cocaine reward contexts. (**A**) Diagrammatic illustration of the calcium imaging recording experiment during pre– and post–cocaine place conditioning. “S,” conditioning with saline injection; “C,” conditioning with cocaine injection. (**B**) Bar graph showing the time spent in cocaine chamber during pre– and post–cocaine place conditioning. (**C**) Top: Representative calcium transients of neurons from one mouse during staying in saline (S)– or cocaine (C)–coupled chambers. Both the cocaine chamber–excited neurons (orange) and cocaine chamber–inhibited neurons (blue) are shown. Bottom: Average calcium traces of the above cocaine chamber–excited neurons (orange) and cocaine chamber–inhibited neurons (blue). (**D**) Top: Pie chart showing the fraction of cells that were excited by (orange), inhibited by (blue), or had no response to (gray) staying in a cocaine-associated chamber during preconditioning (left) or postconditioning (right). Bottom: Bar graphs showing the percentage of neurons that were excited (orange) or inhibited (blue) when staying in a cocaine-associated chamber. (**E**) Calcium transient frequency of cocaine chamber–excited neurons (left, *n* = 16) and cocaine chamber–inhibited neurons (right, *n* = 62) during mice staying in saline or cocaine chambers. (**F**) Left: Average calcium transient frequency of cocaine chamber–excited neurons before and after entering cocaine chamber. Right: Quantification of calcium transient frequency in 5-s windows before and after entry. (**G**) Left: Average calcium transient frequency of cocaine chamber–inhibited neurons before and after entering cocaine chamber. Right: Quantification of calcium transient frequency in 5-s windows before and after entry. Data in (B), (D), (G), and (H) are presented as means ± SEM; shaded areas in (C) represent SEM. The *P* values are calculated on the basis of statistical tests in table S1. **P ≤ 0.01; ****P ≤ 0.0001; ns, *P* > 0.05.

Next, we analyzed the dynamics of *Tac2* neuronal activity during cocaine reward contextual association. To this end, we applied single-cell calcium imaging to a cocaine conditioned place preference (CPP) test (Fig. 2A). In the preconditioning session, mice were allowed to freely explore the two chambers with differential contextual patterns while the calcium signal was being recorded. In the conditioning sessions, mice were given alternate saline and cocaine (15 mg/kg) injections (6 hours apart) and were confined to a specific chamber paired with saline (namely, saline chamber) or cocaine (namely, cocaine chamber). In the postconditioning session, mice were again allowed to freely explore the two chambers while recording calcium signal (Fig. 2A). As expected, mice exhibited a significant preference to the cocaine chamber after conditioning (Fig. 2B), indicating the establishment of a cocaine-related contextual memory. After extracting the calcium signal traces, and corresponding calcium signal transients of individual neurons in preconditioning and postconditioning sessions, we compared the signal traces or transient frequencies during the periods when mice are in saline-coupled chamber or cocaine-coupled chamber (staying time > 5 s). A neuron was identified as cocaine-associated context encoding (CACE) neuron if the average calcium transient frequencies were significantly altered when mice stayed in the two different chambers. We found that CACE neurons exhibit either higher or lower calcium signal in the cocaine chamber when compared to that in the saline chamber (Fig. 2, C and E). In the preconditioning session, we observed a comparable proportion of recorded neurons that showed higher (21 of 367, or 5.7%) or lower (24 of 367, or 6.5%) activity in the cocaine chamber (Fig. 2D), suggesting that *Tac2*^+^ neurons do not distinguish the two chambers before cocaine conditioning. However, after cocaine conditioning, the *Tac2*^+^ neurons that showed reduced activity in the cocaine chamber (62 of 370, or 16.8%) were significantly increased, while those showing elevated activity in the cocaine chamber were slightly decreased (16 of 370, or 4.3%) (Fig. 2D). To further validate these findings, we analyzed calcium activity traces when mice stayed in the saline chamber or cocaine chamber. In the preconditioning session, a small and comparable proportion of neurons altered their neuronal activity when staying in the cocaine chamber (12 of 367, or 3.3% excited; 10 of 367, or 2.7% inhibited). In the postconditioning session, a substantial increase in the CACE neurons with a larger proportion of cocaine chamber–inhibited neurons was observed (40 of 370, or 10.8% excited; 59 of 370, or 16.0% inhibited) (fig. S2A). We found that the CACE neurons emerging after cocaine conditioning rapidly changed their activity when mice entered the other chamber and that calcium traces and corresponding transients of CACE neurons from a representative mouse showed the two groups of neurons with opposite response patterns when the animal stayed in the two chambers (Fig. 2C), suggesting that these neurons could distinguish the environment associated with saline and cocaine after conditioning. Moreover, by focusing on the short period around the mice entering the cocaine chamber (−5 to 5 s, regarding the entering as 0 s), we found that the neuronal activity of the cocaine chamber–excited neurons (neurons with higher calcium signal in cocaine chamber) increased rapidly when mice entered the cocaine chamber, while the cocaine chamber–inhibited neurons (neurons with higher calcium signal in cocaine chamber) decreased their activity when mice entered the cocaine chamber (Fig. 2, F and G). This result suggested that cocaine context–responsive neurons might also be involved in the decision-making of chamber entries. Overall, these results suggest that cocaine conditioning modulates *Tac2* neuronal activities that are related to cocaine reward contextual associations.

### NAc Tac2^+^ neurons bidirectionally regulate cocaine-induced place preference

**Fig. 3.**
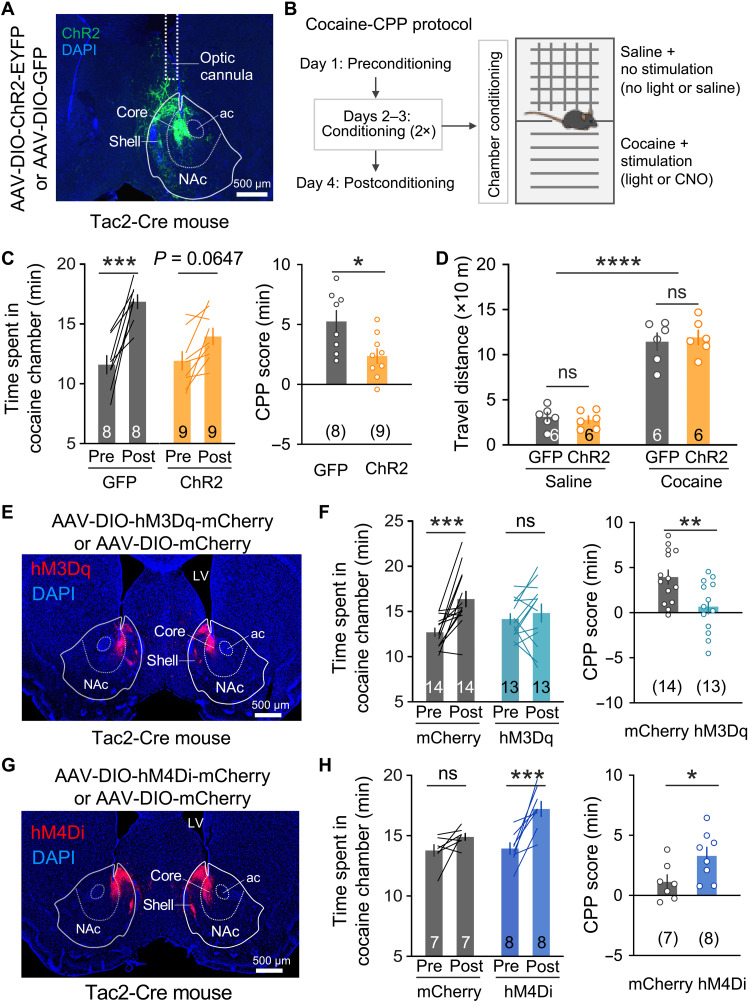
NAc *Tac2*^+^ neurons bidirectionally regulate cocaine reward in the cocaine-CPP test. (**A**) Viral expression of ChR2-EYFP (enhanced yellow fluorescent protein) and optic cannula placement in NAc. DAPI, 4′,6-diamidino-2-phenylindole; ac, anterior commissure. (**B**) Illustration of the two-chamber cocaine-CPP paradigm. Details are described in the “Cocaine-CPP” section. (**C**) Cocaine-CPP with optogenetic excitation of *Tac2*^+^ neurons. Left: Time spent in the cocaine-paired chambers pre- and postconditioning. Right: The CPP scores were calculated by subtracting the time spent in the preconditioning phase from the time spent in the postconditioning phase. (**D**) Cumulative distance traveled in the 30-min posttreatment period. Mice received saline or cocaine injections and were concurrently given laser stimulation [light pattern: 5 × (3 min on and 3 min off)]. (**E**) Viral expression of hM3Dq-mCherry in the NAc. (**F**) Cocaine-CPP with chemogenetic excitation of *Tac2*^+^ neurons. Left: Time spent in the cocaine-paired chambers pre- and postconditioning. Right: The CPP scores were calculated by subtracting the time spent in the preconditioning phase from the time spent in the postconditioning phase. (**G**) Viral expression of hM4Di-mCherry in the NAc. (**H**) Cocaine-CPP with chemogenetic inhibition of *Tac2*^+^ neurons. Left: Time spent in the cocaine-paired chambers pre- and postconditioning. Right: The CPP scores were calculated by subtracting the time spent in the preconditioning phase from the time spent in the postconditioning phase. Data in (C), (D), (F), and (H) are presented as means ± SEM; scale bars of (A), (E), and (G) are indicated in the figures. The *P* values are calculated on the basis of statistical tests in table S1. *P ≤ 0.05; **P ≤ 0.01; ***P ≤ 0.001; ****P ≤ 0.0001; ns, *P* > 0.05.

Because both acute cocaine administration (Fig. 1) and cocaine place conditioning (Fig. 2) could modulate the activity of *Tac2*^+^ neurons, we hypothesized that they are involved in regulating cocaine-related behaviors. To test this hypothesis, we unilaterally injected adeno-associated virus (AAV) vectors expressing light-gated cation channel channelrhodopsin (ChR2) or green fluorescent protein (GFP) into the NAc of two cohorts of *Tac2*-Cre mice and implanted optical cannulas above the viral injection sites for light delivery (Fig. 3A). After confirming the efficacy of optogenetic activation of *Tac2*^+^ neurons with c-Fos induction (fig. S3A) and post-histological verification of optic cannulas locations (fig. S3B), we examined whether *Tac2* neuronal activation affects cocaine reward in a CPP model. Specifically, in the preconditioning session, mice were allowed to freely explore two interconnected chambers with different tactile cues to establish a baseline preference. Then, in the following conditioning session, mice were first conditioned with saline plus no-laser stimulation in one chamber for 30 min and then conditioned with cocaine (15 mg/kg, intraperitoneally) plus laser stimulation in the other chamber for 30 min (Fig. 3B). In the postconditioning session, the preference of the mice to the two chambers was tested again. We found that cocaine conditioning significantly increased the preference of the GFP-expressing mice to the cocaine/laser chamber. However, this preference was largely attenuated by *Tac2* neuronal activation in the ChR2-expressing mice (Fig. 3C), suggesting that activation of *Tac2*^+^ neurons attenuated the formation of cocaine CPP. Recent studies have revealed that some D1 MSNs in the NAc encode an aversive signal (*18*), raising the possibility that activating *Tac2*^+^ neurons may confer such negative valence, thus attenuating the rewarding effect of cocaine in the CPP tests. However, activation of *Tac2*^+^ neurons has no significant effect on real-time place preference (fig. S3C), suggesting that the function of *Tac2*^+^ neurons in regulating cocaine CPP could not be explained by adding an extra reward/aversive effect to the drug. Notably, we found that manipulation of *Tac2* neuronal activity affected neither basal locomotion in open arena (fig. S3D), time spent in the center area of open-field arena (fig. S3E), nor cocaine-induced hyperlocomotion (Fig. 3D). In addition, although previous studies have established a role for NAc D1 MSN in feeding (*44*) and anxiety (*45*), we found that *Tac2* neuronal activation affected neither food intake nor elevated plus maze test (fig. S3, F and G), which supports the existence of functionally distinct D1 MSN subtypes.

To further confirm the above observation, we performed another set of cocaine-CPP experiments using chemogenetic tools. To this end, we introduced Cre-dependent AAV encoding a modified human M3 muscarinic receptor (hM3Dq) into NAc of *Tac2*-Cre mice (Fig. 3E). Administration of clozapine-*N*-oxide (CNO; 2 mg/kg, intraperitoneally) resulted in c-Fos induction in the hM3Dq-expressing neurons, but not in the mCherry-expressing neurons (fig. S4A), confirming the efficacy and specificity of chemogenetic activation of *Tac2*^+^ neurons. We next performed the cocaine-CPP test, in which mice were conditioned with cocaine plus CNO in one chamber, while with saline in the other chamber. Similar to the optogenetic activation result, chemogenetic activation of the *Tac2*^+^ neurons completely abolished cocaine-induced CPP (Fig. 3F). Previous studies have demonstrated that optogenetic activation of pan-D1 MSNs in NAc promoted cocaine CPP (*10*). Unexpectedly, here, we found that activation of *Tac2*^+^ neurons, a subtype of D1 MSN, attenuated cocaine reward behavior. To further confirm our results, we examined the effect of *Tac2* neuronal inhibition on cocaine CPP. To this end, we injected chemogenetic inhibitory vector AAV-DIO-hM4Di-mCherry into NAc of *Tac2*-Cre mice (Fig. 3G) and found that CNO treatment (5 mg/kg, intraperitoneally) significantly decreased c-Fos induction in the hM4Di-expressing neurons following cocaine exposure (fig. S4B), indicating successful inhibition of the neuronal activity of the *Tac2*^+^ neurons. To test the effect of *Tac2* neuronal inhibition on cocaine-CPP, we applied a subthreshold cocaine CPP paradigm, in which mice were conditioned with a lower dose of cocaine (10 mg/kg, intraperitoneally) in a shorter conditioning session (15-min per session) (*10*, *46*). We found that CNO treatment (5 mg/kg, intraperitoneally) resulted in a significantly higher cocaine CPP in the hM4Di-expressing mice when compared to mCherry-expressing mice (Fig. 3H), suggesting that suppression of *Tac2* neuronal activity enhanced the reward effect of cocaine. Similar to the optogenetic activation of the *Tac2*^+^ neurons, chemogenetic-mediated bidirectional manipulation of *Tac2*^+^ neurons affected neither basal locomotion nor cocaine-induced hyperlocomotion (fig. S4, C and D), further supporting the functional specificity of the *Tac2*-expressing D1 MSN subtype in regulating different cocaine-related behaviors. Collectively, these results demonstrate that *Tac2*-expressing D1 MSNs play critical roles in regulating cocaine reward behaviors.

### NAc Tac2^+^ neurons regulate cocaine intravenous self-administration behavior

**Fig. 4.**
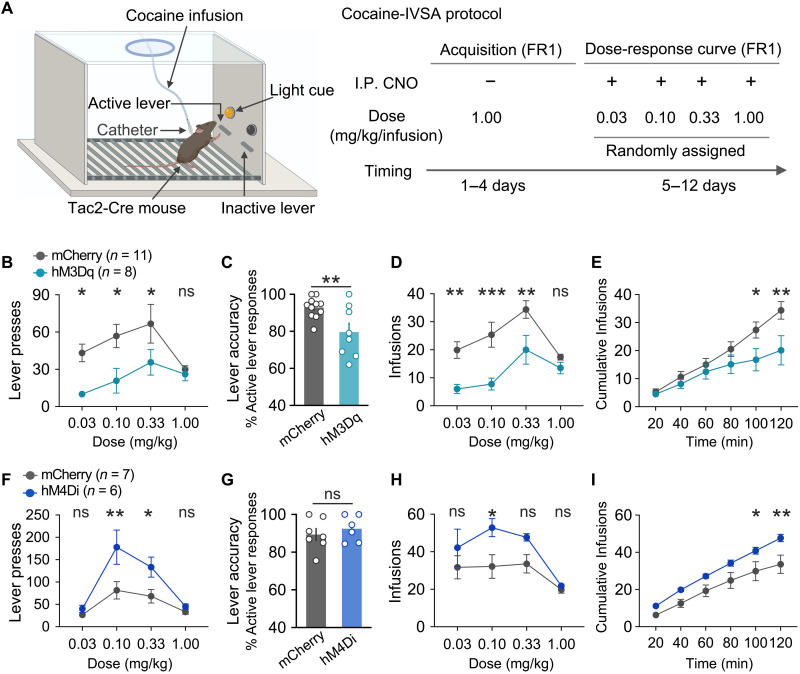
NAc *Tac2*^+^ neurons bidirectionally regulate cocaine reward in the cocaine-IVSA test. (**A**) Diagrammatic illustration of the cocaine intravenous self-administration paradigm (cocaine-IVSA). Mice were trained to press the lever to get cocaine infusion; pressing the active lever is followed by intravenous cocaine infusion, while pressing the inactive lever yields no outcome. The behavioral training includes acquisition phase and dose-response curve in the maintenance phase. Mice received CNO injection (2 mg/kg for the hM3Dq group and 5 mg/kg for the hM4Di group) 20 min before they were placed into the self-administration chamber with access to cocaine at doses of 0.03, 0.1, 0.3, or 1 mg/kg per infusion. (**B** to **E**) Cocaine-IVSA with chemogenetic excitation of *Tac2*^+^ neurons. (B) Numbers of lever presses. (C) Lever accuracy under the dose of 0.33 mg/kg per infusion. (D) Numbers of infusions. (E) Cumulative cocaine infusion time courses under the dose of 0.33 mg/kg per infusion. (**F** to **I**) Cocaine-IVSA with chemogenetic inhibition of *Tac2*^+^ neurons. (F) Numbers of lever presses. (G) Lever accuracy under the dose of 0.33 mg/kg per infusion. (H) Numbers of infusions. (I) Cumulative cocaine infusion time courses under the dose of 0.33 mg/kg per infusion. Data in (B) to (I) are presented as means ± SEM. The *P* values are calculated on the basis of statistical tests in table S1.*P ≤ 0.05; **P ≤ 0.01; ***P ≤ 0.001; ns, *P* > 0.05.

Having established a critical role of the NAc *Tac2*^+^ neurons in regulating cocaine reward behavior, we further examined the function of *Tac2*^+^ neurons in cocaine intravenous self-administration (cocaine-IVSA), a clinically relevant drug addiction model. In this model, mice with indwelling jugular catheters were trained to press the active lever to self-administrate cocaine (1 mg/kg per infusion) in the operant chamber, while pressing the inactive lever would yield no outcome (Fig. 4A). To evaluate the effect of *Tac2* neuronal activation, we compared the drug-taking behavior between hM3Dq- and mCherry-expressing mice (different cohorts from the ones used in the above cocaine-CPP test). Following 4 days of training, mice showed significantly higher numbers of active lever presses than inactive lever presses (fig. S5, A and B), suggesting successful acquisition of cocaine self-administration behavior. Then, we compared the cocaine taking between the two groups under different cocaine doses (0.03, 0.1, 0.3, and 1 mg/kg per infusion) following CNO administration (Fig. 4B). We found that the hM3Dq group showed a significantly fewer number of lever presses (Fig. 4B), reduced lever accuracy (Fig. 4C), and fewer cocaine infusions (Fig. 4, D and E), compared to the control mCherry-expressing group, suggesting that *Tac2* neuronal activation suppressed the cocaine self-administration behavior. To evaluate the effect of *Tac2* neuronal inhibition, we next compared the drug-taking behavior between hM4Di- and mCherry-expressing mice (the same cohort of mice used in Fig. 3 was used, but the two experiments were performed with more than 1 week of gap time to avoid potential CNO-induced changes) using the same cocaine-IVSA paradigm. After acquiring stable self-administration behavior (fig. S5, C and D), mice were tested under different cocaine doses following CNO treatment. We found that CNO treatment resulted in a significantly higher number of lever presses (Fig. 4F), comparable lever accuracy (Fig. 4G), and significantly lower number of cocaine infusions (Fig. 4, H and I) in hM4Di-expressing mice, compared to the control mCherry-expressing group, suggesting that *Tac2* neuronal inhibition promoted cocaine self-administration. Collectively, the bidirectional manipulation of *Tac2*^+^ neurons indicates that *Tac2* neuronal activity negatively regulates the contingent cocaine-taking behavior.

### The NAc to LH projection of Tac2^+^ D1 MSNs regulates cocaine reward memory

**Fig. 5.**
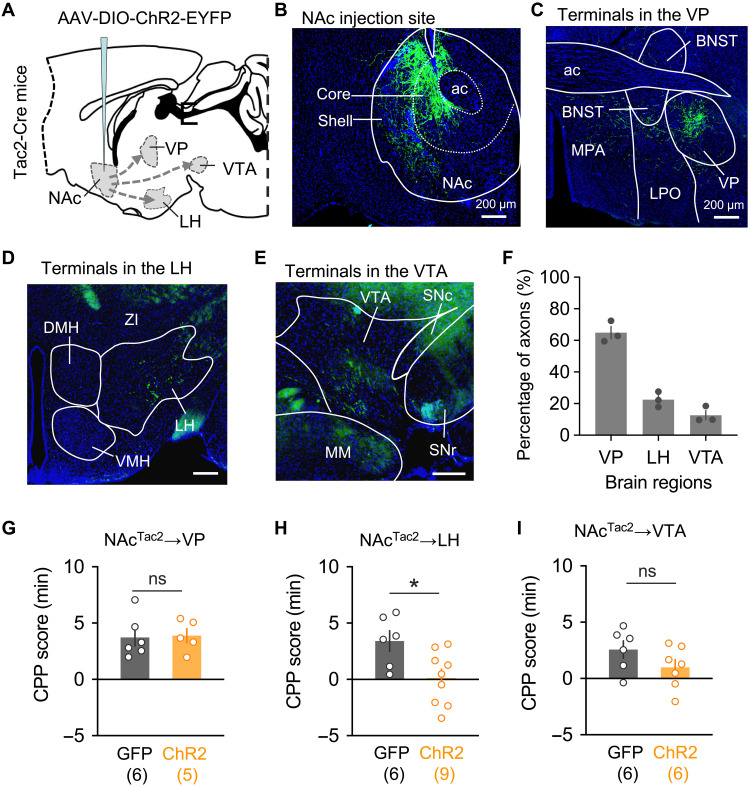
The *Tac2*^+^ NAc→LH pathway modulates cocaine reward behavior. (**A**) Diagram of antegrade tracing of the NAc *Tac2*^+^ neurons with ChR2-EYFP. (**B**) AAV-DIO-ChR2-EYFP expression in the NAc injection site. (**C** to **E**) Detection of the ChR2-EYFP signals in the nerve terminals in ventral pallidum (VP) (C), lateral hypothalamus (LH) (D), and ventral tegmental area (VTA) (E). (**F**) Percentages of axon terminal fluorescence signal in VP, LH, and VTA. (**G** to **I**) Optogenetic activation of *Tac2*^+^ NAc→LH projection (H), but not NAc→VP (G) and NAc→VTA projections (I), reduced the cocaine-CPP score. Data in (F) to (I) are presented as means ± SEM; scale bars of (B) to (E) are indicated in the figures. BNST, bed nuclei of the stria terminalis; MPO, medial preoptic area; LPO, lateral preoptic area; ZI, zona incerta; DMH, dorsal medial hypothalamus; VMH, ventral medial hypothalamus; IPN, interpeduncular nucleus; MM, medial mammillary nucleus; SNr, substantia nigra, reticular part; SNc, substantia nigra, compact part. The *P* values are calculated on the basis of statistical tests in table S1. *P ≤ 0.05; ns, *P* > 0.05.

Using calcium recording and neuronal activity manipulation, we have established a causal relationship between *Tac2* neuronal activity and cocaine-associated behaviors (CPP and IVSA). We next explored how *Tac2^+^* neurons might execute their functions. Given *Tac2* gene–encoded neuropeptide neurokinin B (NKB), which has been implicated in various neurological processes, including social stress (*47*) and fear memory (*48*), we asked whether the *Tac2*-encoded neuropeptide is involved. To this end, we used a genetic approach to specifically knock down *Tac2* mRNA in the NAc region and then tested cocaine-related behaviors. We packaged AAV construct expressing a previously tested Tac2–short hairpin RNA (shRNA) (*47*) and bilaterally injected into the NAc region of wild-type mice. After confirming knockdown efficiency (fig. S6, A and B), mice were subjected to cocaine-related behavior tests. We found that *Tac2* knockdown affected neither cocaine-induced place preference (fig. S6C) nor contingent cocaine taking (fig. S6, D to I). We next investigated whether *Tac2^+^* cells innervate downstream neurons through direct neuronal connection. To this end, we first performed ChR2-mediated antegrade tracing (Fig. 5, A and B), which revealed multiple projection targets of *Tac2*^+^ neurons, including ventral pallidum (VP), lateral hypothalamus (LH), and ventral tegmental area (VTA) (Fig. 5, C to F). To further determine which of these projections are involved in regulating cocaine reward behavior, we injected AAV-DIO-ChR2 to NAc and implanted optic cannulas into VP, LH, and VTA, respectively, for selective optogenetic activation of each of the projections upon behavioral tests. We found that optogenetic activation of the NAc to LH projection, but not the NAc to VP or NAc to VTA projection, significantly reduced the cocaine-CPP (Fig. 5D). These results suggest that NAc *Tac2*^+^ neurons regulate cocaine reward memory mainly through their projection to LH.

## DISCUSSION

Despite the demonstration of the versatile roles of the D1 and D2 MSN in regulating addiction-related behavior, the underlying mechanism remains largely unknown. Recent studies using single-cell profiling, in vivo calcium imaging, and cell type/circuitry-specific manipulation have revealed a rich striatal neuronal diversity in their gene expression, spatial location, neural connection, and function (*31*, *38*, *49*), supporting the notion that molecularly distinct MSN subtypes in the striatum may contribute, at least partially, to its functional complexity. However, this notion has not been directly tested in the context of drug addiction. Here, we report the functional characterization of a *Tac2*-expressing D1 MSN subtype. Our results suggest that these *Tac2*^+^ neurons play an “unconventional” role in regulating cocaine reward and addiction-related behavior. First, we found that acute cocaine administration represses the overall activity of *Tac2*^+^ neurons (Fig. 1), and cocaine conditioning induces neuronal activity changes with more *Tac2*^+^ neurons inhibited than activated in cocaine-associated context (Fig. 2), which is opposite to the conventional view of D1 MSNs in the direct/indirect pathway model and the in vivo recordings on pan-D1 MSNs (*50*, *51*). Second, through bidirectional activity manipulation, we showed that the *Tac2*^+^ neurons negatively regulate cocaine reward (Fig. 3) and voluntary drug self-administration (Fig. 4), which is in contrast to the findings made when pan-D1 neurons were manipulated (*10*). Notably, this phenomenon could not be simply attributed to conveying negative valence by *Tac2*^+^ neuronal activation (fig. S3E). Last, through projection-specific optogenetic intervention, we demonstrated that the NAc to LH projection, but not the NAc to VP or NAc to VTA projection, of *Tac2*^+^ neurons underlies its role in regulating drug reward (Fig. 5). Collectively, our study establishes a critical role of a molecularly defined D1 MSN subtype in regulating cocaine addiction, which is unaccounted for in the conventional model.

Our findings significantly contribute to the understanding of the role of NAc in drug addiction and have important clinical implications. First, the *Tac2*-expressing D1 MSN subtype represents an evolutionally conservative D1 MSN subtype in both rodent and primate (*31*, *39*), and the discovery that *Tac2 -*expressing NAc D1 MSNs negatively regulate cocaine reward and addiction behaviors is a conceptual advance over the conventional dichotomy model, which suggests that D1 MSNs positively regulate addiction behaviors (*10*, *40*). Given that NAc D1 MSNs are highly heterogeneous and can be further classified into multiple molecularly and spatially distinct subtypes (*29*–*31*), our results call for functional characterizations of these neuron subtypes to gain more detailed knowledge with regard to the function of the NAc D1 MSNs. Similar studies should also be carried out for the NAc D2 MSNs as they exhibit a similar heterogeneity and complexity (*29*, *31*). We believe that the functional characterization of these D1 and D2 subtypes should shed light on the cellular and molecular mechanisms underlying the functional complexity of NAc. Second, our discovery that the function of *Tac2*^+^ neurons in regulating addiction-related behavior is largely mediated by the NAc to LH projection, but not the NAc to VP or NAc to VTA projection, further illustrates the complexity in behavioral regulation. This indicates that the same molecularly defined neuron subtype can have different functions depending on its projection. The NAc to LH projection of pan-D1 MSNs had been previously shown to negatively regulate natural food (*2*, *44*), alcohol (*17*), and opiate reward behaviors (*46*). Our discovery raises the intriguing question of whether the NAc *Tac2*–expressing D1 MSN neurons projecting to LH regulate all these reward-related behaviors or are independently regulated by different NAc D1 MSN subtypes that all projected to LH. Further studies are needed to distinguish between these two possibilities. Last, our findings also have potential clinical implications. Although the involvement of NAc in motivational behaviors of both drugs and natural rewards (*25*, *26*, *40*) has been shown, the molecularly and functionally defined MSN subtypes beyond the pan-D1 and pan-D2 neurons in cocaine addiction have not been identified and characterized, making therapeutical intervention difficult. Here, we applied a series of addiction-related behaviors, including the cocaine-induced place preference and the clinically relevant IVSA model, to demonstrate the role of the molecularly defined NAc *Tac2*–expressing D1 neurons in suppressing cocaine addiction behavior. The conservation of the *Tac2* D1 MSN subtype in rodent and primate and their critical function in addiction-associated behaviors raise the intriguing possibility that targeted stimulation of the NAc *Tac2*–expressing neurons might be a potential strategy for treating drug addicts.

## MATERIALS AND METHODS

### Mice

All experiments were conducted in accordance with the National Institutes of Health *Guide for Care and Use of Laboratory Animals* and approved by the Institutional Animal Care and Use Committee of Boston Children’s Hospital and Harvard Medical School. The *Tac2*-Cre knock-in mouse line was a gift from Q. Ma at Dana-Farber Cancer Institute and Harvard Medical School. The *Tac2*-Cre line was backcrossed to the C57BL/6N background. Mice were group-housed under controlled temperature (22° to 25°C) conditions in a 12-hour light-dark cycle (light time, 7:00 to 19:00) with ad libitum chow food (4% fat specific pathogen–free rodent feed) and water. All experiments were performed using male adult mice (8 to 16 weeks), and the behavioral experiments were all conducted during the light cycle.

### AAV vectors

The following AAV vectors (with a titer of >10^12^) were purchased from the vector core at the University of North Carolina at Chapel Hill (UNC Vector Core): AAV5-EF1a-DIO-hChR2(H134R)-EYFP, AAV5-EF1a-DIO-EYFP, and AAV-DJ-EF1aDIOGCaMP6m. The following AAV vectors were from Addgene: AAV5-hSyn-DIO-hM3D(Gq)-mCherry (no. 44361), AAV5-hSyn-DIO-hM4D(Gi)-mCherry (no. 44362), and AAV5-hSyn-DIO-mCherry (no. 50459).

### Tac2 shRNA knockdown

shRNA for mouse *Tac2* gene (NM_009312.2) was selected on the basis of literature (*50*), and the oligonucleotides encoding *Tac2* shRNA were as follows: 5′-gatccgCCGCCTCAACCCCATAGCAATTAgaagcttgTAATTGCTATGGGGTTGAGGCttttttt-3′ and 3′-gcGGCGGAGTTGGGGTATCGTTAATcttcgaacATTAACGATACCCCAACTCCGaaaaaaagatc-5′. The oligonucleotides were cloned into the AAV vector backbone AAV-shRNA-Ctrl (Addgene, no. 85741), and both AAV-Tac2-shRNA and AAV-shRNA-Ctrl constructs were packaged by the Viral Core of Boston Children’s Hospital. The AAV viruses were injected into NAc regions, and knockdown efficiency was evaluated by *Tac2* RNA fluorescence in situ hybridization (FISH).

### FISH and IF staining

Mice were transcardially perfused with phosphate-buffered saline (PBS) followed by 4% paraformaldehyde. Brains were then placed in a 30% sucrose solution for 2 days. The brains were frozen in optimal cutting temperature embedding medium, and 16-μm (for FISH) or 40-μm [for immunofluorescence (IF)] coronal sections were cut with a vibratome (Leica, no. CM3050 S). For FISH experiments, the slices were mounted on SuperFrost Plus slides and air-dried. The multicolor FISH experiments were performed following the instructions of the RNAscope Fluorescent Multiplex Assay (ACD Bioscience). For IF, cryostat sections were collected and incubated overnight with blocking solution (1× PBS containing 5% goat serum, 2.5% bovine serum albumin, and 0.1% Triton X-100), then treated with the following primary antibodies, and diluted with blocking solution for 1 day at 4°C: rabbit anti–c-Fos (1:2000; Synaptic Systems, no. 226003), chicken anti-GFP (1:2000; Aves Labs, no. GFP-1010), and chicken anti-mCherry (1:2000; Novus Biologicals, no. NBP2-25158). Samples were then washed three times with washing buffer (1× PBS containing 0.1% Triton X-100) and incubated with the Alexa Fluor–conjugated secondary antibodies for 2 hours at room temperature. The sections were mounted and imaged using a Zeiss LSM800 confocal microscope or the Olympus VS120 Slide Scanning System.

### Image processing and quantification

NAc *Tac2^+^* axonal projections were quantified by measuring the fluorescence signal intensity of targeted regions. Serial 40-μm coronal sections (spaced 120 μm apart) from *Tac2*-Cre::EF1a-DIO-hChR2(H134R)-EYFP–expressing mice were collected and imaged with the VS120 Slide Scanning System (Olympus). Images collected were first preprocessed using OlyVIA software (Olympus) and then imported into Fiji software with background subtraction and brightness and contrast adjustment. Regions of interest were selected on the basis of boundaries of brain regions defined by the mouse brain atlas (*52*). Fluorescence signal intensities were measured using Fiji software.

### Stereotaxic brain surgeries

The AAV vectors were injected through a pulled glass pipette and a nanoliter injector (Nanoject III, Drummond Scientific, 3-000-207). The injection was performed using a small-animal stereotaxic instrument (David Kopf Instruments, model 940) under general anesthesia by isoflurane (0.8 liters/min; isoflurane concentration, 1.5%) in oxygen. A feedback heater was used to keep mice warm during surgeries. Mice were allowed to recover in a warm blanket before they were transferred to housing cages for 2 to 4 weeks before the behavioral evaluation was performed. For chemogenetic experiments, 0.1 to 0.15 μl of AAV vector were bilaterally delivered into target regions. For optogenetic experiments, following viral injection, the fiber-optic cannulas (200 μm in diameter; Inper Inc., China) were implanted 0.3 mm above the viral injection site and were secured with dental cement (Parkell, no. S380). The coordinates of viral injection sites are based on previous literature (*52*) as follows: NAc [+1.2 mm Anterior-Posterior (AP), ± 0.6 mm Medial-Lateral (ML), −4.5 mm Dorsal-Ventral (DV)], VP (+0.1 mm AP, ± 1.4 mm ML, −5.0 mm DV), LH (−1.3 mm AP, ± 1.1 mm ML, −5.0 mm DV), and VTA (−3.2 mm AP, ± 0.6 mm ML, −4.8 mm DV). For the calcium recording experiments, 0.3 to 0.4 μl of viruses were injected into the NAc region; 4 to 5 weeks later, a GRIN lens (0.6 mm diameter, 7 mm length; Inscopix) was implanted 0.3 mm above the viral injection site and was secured with dental cement. One week later, a baseplate that holds the miniature microscope (nVoke 1.0, Inscopix) was attached to the lens.

### Calcium activity imaging by a miniature endomicroscope

Two weeks after GRIN lens implantation surgery, a baseplate was implanted to mount the miniature microscope and to check the fluorescence signal. Freely moving in vivo imaging can be performed after the nVoke microscope (Inscopix) is attached to the fixed baseplate. All recordings were made at 10 frames per second, light-emitting diode intensities were 0.6 to 1.2 mW, and gain (1.5×) was used. The calcium traces were extracted using Inscopix Data Processing Software (version 1.6, Inscopix). The raw recording videos were cropped, spatially downsampled (2×), spatially filtered (cutoffs: low, 0.005 pixel^−1^; high, 0.500 pixel^−1^), and motion-corrected (using a reference frame from the input movie). The spatial location of individual neurons and their associated calcium activity signals were identified using the extended constrained non-negative matrix factorization algorithm (*53*, *54*) and outputted temporal traces as delta F over noise. The identified neurons were manually checked to exclude overlapping neurons, blood vessels, or fluorescent debris moving in and out of focus. The calcium signal traces were further deconvolved using OASIS (online active set method to infer spikes) methods (*1*) to estimate underlying calcium signal transients. The data matrix containing calcium signal traces and calcium signal transients was subjected to custom MATLAB code for further analysis.

### Calcium imaging during acute saline/cocaine administration

Mice were tested in the home cages and prehabituated in the test room, and they received 3-day saline injection to reduce stress. On day 1, mice were attached with an nVoke microscope 30 min before experiment. Before injection, 10-min baseline was recorded, then mice were intraperitoneally injected with saline, and a 10-min recording was performed after the injection. On day 2, mice were again attached with an nVoke microscope and recorded for 10-min baseline. Then, mice were injected with cocaine (15 mg/kg) intraperitoneally. The postinjection initial phase (0 to 10 min) and decay phase (50 to 60 min) were recorded. We used two datasets of neuronal activity, calcium signal traces over time or calcium signal transients over time, to analyze treatment (saline or cocaine injection)–responsive neurons (excited or inhibited by treatment), and 10-min windows of pre- and postinjection were compared. For saline injection–responsive neurons, saline (−10 to 0 min) and saline (0 to 10 min) responses of the two groups were compared. For cocaine injection–responsive neurons, the initial phase (0 to 10 min) and the decay phase (50 to 60 min) were compared with the preinjection phase (−10 to 0 min). When analyzing the calcium signal transients of 10-min windows pre- and postinjection, we first calculated the “actual transient change” by subtracting the “preinjection transients” from the “postinjection transients.” We then combined transients of the two 10-min windows, the preinjection transients and postinjection transients, to generate “all transients,” and took intertransient intervals of the all transients. We used the permutation method to create 10,000 shuffled distributions of the intertransient intervals, generated “shuffled pretransients” and “shuffled posttransients,” and then calculated their transient number change (“shuffled transient change”) by subtracting shuffled pretransients from shuffled posttransients. We calculated the 1st percentile value and 99th percentile value of the shuffled transient change, and neurons with actual transient change bigger than 99th percentile of the shuffled transient change were considered as injection-excited neurons, and neurons with actual transient change lower than 1st percentile of the shuffled transient change were considered as injection-inhibited neurons. When analyzing the calcium signal traces of 10-min windows, the traces (dF over noise) within 10-min windows were *z*-scored and divided into ten 1-min bins, and traces of each bin were averaged. The treatment-responsive neurons were determined by comparing the averaged traces pre- and postinjection using the Wilcoxon signed-rank test. Neurons with significantly higher (*P* < 0.05) values of postinjection traces were classified as injection-excited neurons, and those with significantly lower (*P* < 0.05) values of post-injection traces were classified as injection-inhibited neurons.

### Calcium imaging during cocaine-CPP

We used custom-made two-compartment CPP chambers (50 cm × 25 cm × 25 cm, *L* × *D* × *H*) with visual cues: The floor and the walls of one chamber were decorated with black horizontal stripes, and the other chamber was decorated with yellow diagonal stripes. The modified cocaine-CPP consists of three sessions. In the preconditioning session, mice were attached with an nVoke microscope and allowed to explore the two CPP chambers and to concurrently record calcium activity for 15 min. The time spent in different chambers was recorded, the less preferred chamber will be assigned as cocaine chamber and will be used to be conditioned with cocaine injections, and the other chamber will be assigned as saline chambers and will be used to be conditioned with saline injections. Then, in the conditioning session, mice were conditioned for 3 days to saline injections in the saline chamber in the morning and to cocaine injection (15 mg/kg) in the cocaine chamber in the afternoon (6 hours apart). The mice were confined in the chambers for 30 min. Last, the next day following conditioning, in the postcondition recording session, mice were again recorded calcium activity while freely exploring the two chambers for 15 min. The mice trajectories were recorded using a webcam (Logitech) with top view, and the time when the mice stayed or entered the chambers was analyzed using EthoVision XT 11 (Noldus) and was also manually inspected. During the 15-min recording windows of the pre- and postconditioning sessions, mice traveled and stayed between the two chambers, and the calcium signal traces and calcium signal transients were aligned with periods that were staying in the two chambers (cocaine chamber and saline chamber, staying time > 5 s) and entering from one chamber to the other one. A neuron was identified as a CACE neuron when neuronal activity (calcium signal traces or calcium signal transient frequency) in cocaine chamber–staying periods was significantly different from neuronal activity in saline chamber–staying periods. When analyzing the calcium signal traces, we first averaged the trace values of each chamber-staying period and then used Mann-Whitney *U* test to compare the average trace values of multiple saline chamber–staying periods with those in the multiple cocaine chamber–staying periods. When analyzing the calcium signal transients, we first calculated transient frequency of each chamber-staying period by dividing staying time into numbers of transients, and then we used Mann-Whitney *U* test to compare the transient frequencies. Neurons with significantly higher (*P* < 0.05) transient frequency during cocaine chamber–staying periods were classified as cocaine chamber–excited neurons, and those with significantly lower (*P* < 0.05) transient frequency during cocaine chamber–staying periods were classified as cocaine chamber–inhibited neurons.

### Behavioral assays

Mice were habituated in the testing environment for at least 3 days before being subjected to behavioral tests to avoid stress. To manipulate the activity of NAc *Tac2*–expressing neurons by chemogenetics, mice were intraperitoneally injected with CNO (Cayman, no. 16882) at 2 mg/kg (for hM3Dq) or 5 mg/kg (for hM4Di) 20 min before behavioral tests. To manipulate the activity of NAc *Tac2*–expressing neurons by optogenetics, the 473-nm laser power was adjusted to 8 to 10 mW at the optic fiber tip, and the laser stimulation protocols were indicated in the figure legends. For all experiments, mice with signs of infection/bleeding/unhealthy conditions after the surgeries were excluded for behavioral tests, and mice with missed viral injection or implantation, based on brain atlas, were not included in experimental analyses.

#### Open-field test

The experiment was conducted in an eight-chamber activity monitor system (Med Associates). Each test chamber (ENV-510 boxes) consists of a 27 cm × 27 cm square base and 24-cm walls. For optogenetic experiments, the total duration of the behavioral test was 15 min, which was divided into three 3 × 5-min epochs (with laser off, on, and off, respectively). The laser pattern is 473 nm, 20 Hz, and 10-ms duration. Activity Monitor software (Med Associates) was used to generate trajectory maps. The distance traveled in the arena and time spent in the center part of the arena were recorded and analyzed. After each test session, the chambers were cleaned thoroughly with 20% ethanol.

#### Cocaine-induced locomotion

The basal level of locomotion and cocaine-induced change of locomotor activity were assessed in the open-field arena similar to the open-field test. For chemogenetic experiments, 20 min after administration of either saline or CNO, mice were individually placed in the center of the test chamber, triggering the infrared tracking system to record locomotor activity, as measured by the number of beam breaks. For optogenetic experiments, the total duration of the behavioral test was 30 min, which was divided into three 5 × 6-min epochs (with 3-min laser on and 3-min laser off, respectively). Different groups of mice were balanced across the eight chambers to avoid potential environmental bias.

#### Post-fasted food intake

Mice were first fasted overnight (16 hours) and then individually placed in the home cage, and regular chow pellets (3 g per pellet) were put in the cage; meanwhile, mice received 20-min laser stimulation (4 × 5 min, on-off-on-off), and then the remaining food pellets were collected and food intake was measured.

#### Elevated plus maze

Elevated plus maze (EPM) was used to measure the anxiety effect. Before the EPM test, mice were brought to the testing room for environmental habituation for at least 30 min. The EPM apparatus consisted of an elevated platform (80 cm above the floor), with four arms (each arm is 30 cm in length and 5 cm in width), two opposing closed arms with 14-cm walls, and two opposing open arms. Mice were attached to the fiber-optic patch cord and were individually placed in the center of the EPM apparatus, toward one of the open arms. For optogenetic experiments, the total duration of the behavioral test was 9 min, which was divided into three 3 × 3-min epochs (with laser off, on, and off, respectively). The mice trajectories were tracked, and the time spent in the open arms was analyzed using EthoVision XT11 (Noldus).

#### Real-time place preference

The mice were tested in a two-chamber cage (60 cm × 30 cm × 30 cm) without additional contextual cues. Mice were gently put into the middle of the cage at the beginning of the test. Mice would receive laser stimulation when entering one side of the chamber randomly assigned at the beginning of the experiment; the laser pattern was 473 nm, 20 Hz, 8 mW, and 10-ms duration. Each mouse was tested for 20 min. The laser-coupled side was randomly assigned. Mice tracks were analyzed using EthoVision XT11 (Noldus).

#### Cocaine-CPP

We used a two-compartment CPP chamber with one compartment with grid rod style floor and the other with mesh style floor (Med Associates). Chamber size is 25 cm × 19 cm × 17 cm (*L* × *D* × *H*). Mice trajectories were tracked by infrared photobeam detectors, and the travel distance and the duration the mice spent in the two compartments were recorded. The CPP protocol consists of three sessions that include preconditioning, conditioning, and postconditioning. The baseline preference was measured when mice explore the two chambers for 30 min, and mice that showed a strong basis (<25% preference) were excluded from the experiments.

In the conditioning session, for ChR2 optogenetic experiments, the optic fibers were secured to the cannula before the experiment. The *Tac2*-Cre mice were conditioned for 2 days with no-laser/saline injections in one chamber in the morning and with laser/cocaine (15 mg/kg) injection in the other chamber in the afternoon (6 hours apart). The mice were confined in the chamber for 30 min, and laser stimulation pattern is 5 × 6 min (3 min on and 3 min off, respectively). For hM3Dq chemogenetic experiments, the *Tac2*-Cre mice were conditioned for 2 days to saline/saline injections in one chamber in the morning and to CNO (2 mg/kg)/cocaine (15 mg/kg) injection in the other in the afternoon (30-min confinement, 6 hours apart). For hM4Di chemogenetic experiments, a suboptimal cocaine-CPP paradigm was used (*10*, *46*), where mice received a lower cocaine dose (10 mg/kg) and were conditioned with chambers for 15 min. The day after conditioning, mice were tested for place preference during a 30-min session where they were allowed to freely explore the two chambers. The time spent in the cocaine-coupled chambers was recorded, and the CPP scores were calculated by subtracting the time spent in the preconditioning phase from the time spent in the postconditioning phase.

#### Intravenous catheterization and cocaine self-administration

Mice were implanted with indwelling catheters (Instech Laboratories Inc., PA, USA; catalog no. C20PU-MJV1926) into the right jugular vein under a combination of ketamine (100 mg/mg) and xylazine (10 mg/mg, intraperitoneally) anesthesia. The catheter was then passed subcutaneously to the back and affixed to a vascular access button (Instech Laboratories Inc., PA, USA; catalog no. VABM1B/ 25). Mice were treated with analgesic meloxicam (2 mg/kg, subcutaneousy) before and 24 hours after surgery. To avoid clotting and to maintain patency, catheters were flushed daily with heparin (30 IU/ml). Three to 4 days after surgery, mice were trained to self-administer cocaine intravenously in a modular operant chamber (Med Associates Inc., St. Albans, VT, USA). During the acquisition phase, self-administration was performed under a fixed ratio 1 (FR1) schedule of reinforcement in which an active lever press resulted in a cocaine infusion (1.0 mg/kg per infusion) paired with a tone/cue light conditioned stimulus (30 s). Inactive lever presses had no programmed consequences. Each infusion was followed by a 30-s time-out period, during which further active lever presses were recorded but did not result in additional intravenous infusion. All responses were recorded automatically using a computer interface and software from Med Associates (St. Albans, VT, USA). Each session lasted for a maximum of 2 hours or until 20 infusions were taken. The acquisition was defined as intake of at least six injections within the session and a 3:1 ratio of active to inactive lever press during two consecutive sessions. Following the acquisition, the dose-response curves were tested in the maintenance phase. Mice were intraperitoneally injected with CNO (2 mg/kg for the hM3Dq group and 5 mg/kg for the hM4Di group) before behavioral tests and then were subsequently tested under an FR1 schedule, with different unit doses of cocaine (1.0, 0.33, 0.1, and 0.033 mg/kg per infusion). Dose order was randomly assigned to each animal. Each unit dose was measured for one session, and between the test doses, mice were stabilized with the maintenance dose. Throughout self-administration, the catheter patency was assessed periodically using the intravenous infusion of ketamine. Animals that failed catheter patency test were removed from the study.

### Data analyses and statistics

The order of the animals in the behavioral tests was randomly assigned. Investigators were not blinded to experimental conditions. However, all data were collected and analyzed strictly in the same way. No statistical methods were used to predetermine sample sizes, but our sample sizes were similar to those reported in previous publications. Individual data points are shown in the bar graphs. Statistical *P* values were calculated using Prism 9 (GraphPad) or MATLAB R2019 (MathWorks). Statistical details are indicated in the relevant figure legends. Statistical significance was set as *P* ≤ 0.05.

## Notes

View/request a protocol for this paper from *Bio-protocol*.
