# Supplementary material for: A molecularly defined D1 medium spiny neuron subtype negatively regulates cocaine addiction
Source: Sci Adv. 2022 Aug 12;8(31):eabn3552. doi: 10.1126/sciadv.abn3552 (PMC9374336; doi:10.1126/sciadv.abn3552)
Supplement: Supplementary file 1 — Figs. S1 to S6 Table S1 [file sciadv.abn3552_sm.pdf]

Science Advances
AAAS

Supplementary Materials for

A molecularly defined D1 medium spiny neuron subtype negatively regulates cocaine addiction

Zheng-dong Zhao *et al.*

Corresponding author: Yi Zhang, yzhang@genetics.med.harvard.edu

Sci. Adv. **8**, eabn3552 (2022)
DOI: 10.1126/sciadv.abn3552

This PDF file includes:

Figs. S1 to S6
Table S1

Zhao et. al., Fig. S1

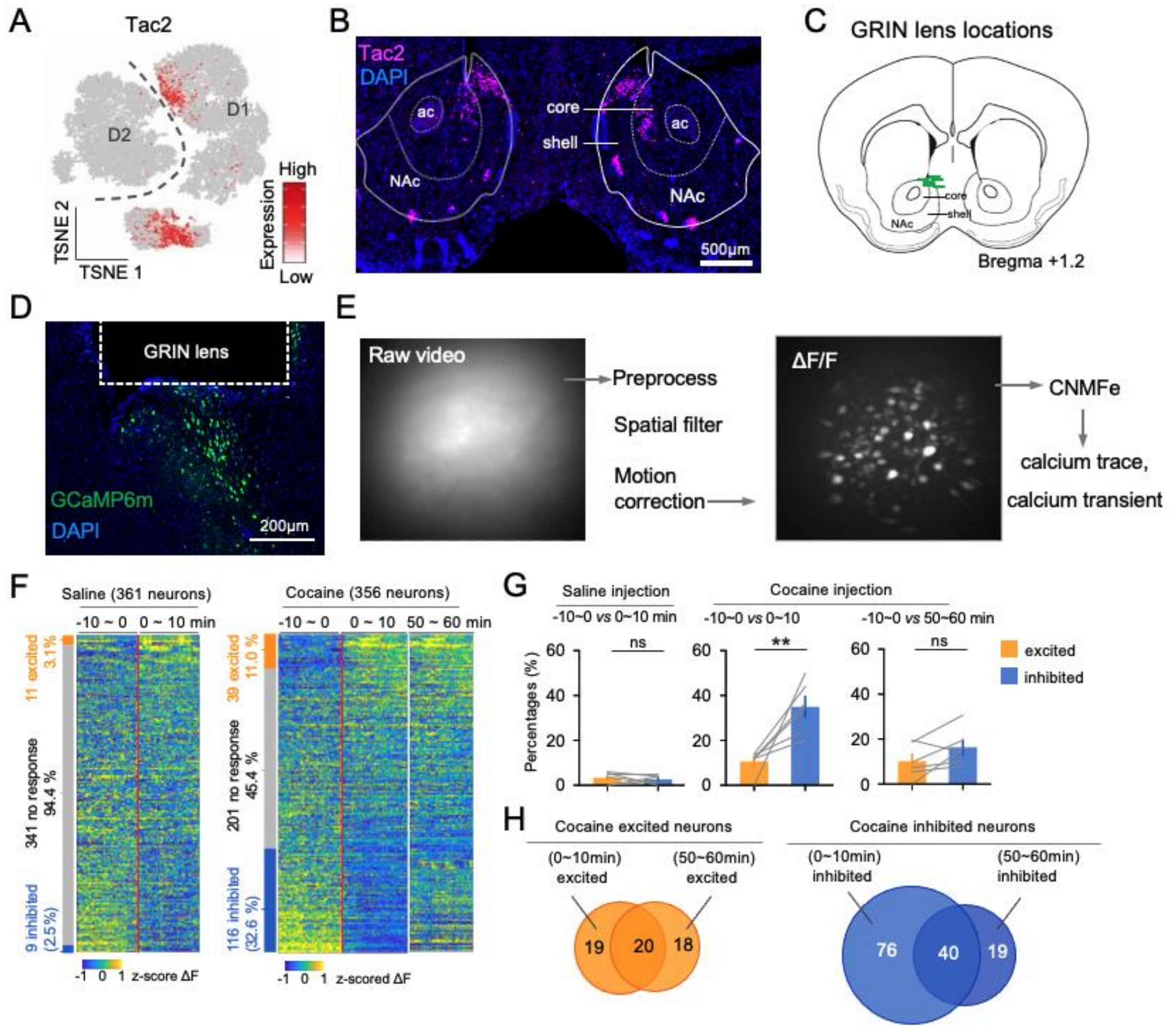

Fig. S1. Single-cell calcium imaging of *Tac2*⁺ neurons following saline/cocaine injection.

A. tSNE plot showing the expression of *Tac2* in a subpopulation of D1-MSN in NAc. The expression level is color-coded.

B. RNA *in situ* hybridization showing *Tac2* expression in the medial part of the NAc shell.

C. A diagram indicating the locations of the implanted GRIN lenses (green lines) in the NAc.

D. Viral expression of GCaMP6m and lens location in NAc.

E. Pipeline for calcium signal extraction and cell identification.

F. Heatmap of calcium signal traces of all neurons from 6 mice recorded following saline (left) or cocaine (right) injection. The neurons that are excited (orange), inhibited (blue) and no response (gray) by treatment are shown in the stacked bar graph next to the heatmap.

G. Analysis based on calcium trace. Bar graphs showing the percentages of neurons that were excited (orange) or inhibited (blue) by saline (left) or cocaine (right) administration. (**, $p=0.0024$; ns, $p > 0.05$, paired t-test).

H. Venn diagrams showing the overlap between the initial phase (0-10 min) and late phase (50-60 min) of post-injection of cocaine excited neurons (left panel) or cocaine inhibited neurons (right panel).

Data in (G) are presented as mean ± SEM, scale bars of (B) and (D) are indicated in the figures.

Zhao et. al., Fig. S2

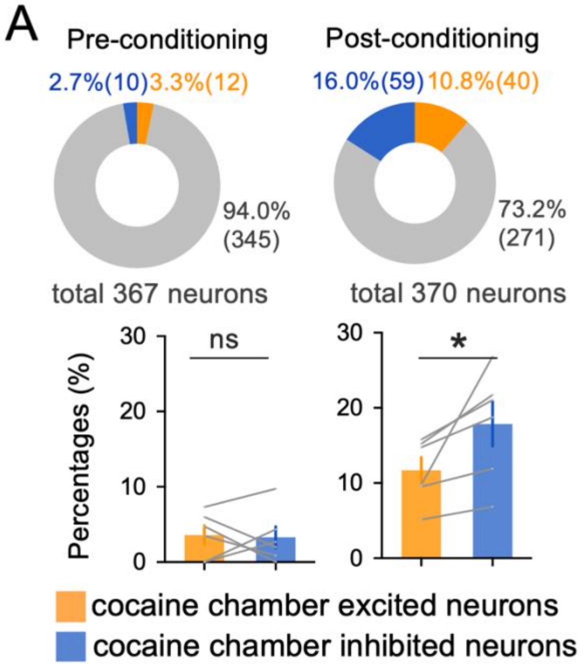

Fig. S2. Percentages of cocaine conditioning responsive neurons in pre- and post-conditioning

A. Upper panel: pie chats showing the fractions of *Tac2*+ neurons that were excited (orange), inhibited (blue), or no response (gray) when staying in a cocaine-associated chamber during pre-conditioning (left) or post-conditioning (right). Bottom panel: bar graphs showing the percentages of neurons that were excited (orange) or inhibited (blue) when staying in a cocaine-associated chamber during pre-conditioning (left) or post-conditioning (right). *, p=0.044, ns, p>0.05, paired t-test. Data are presented as mean ± SEM.

Zhao et. al., Fig. S3

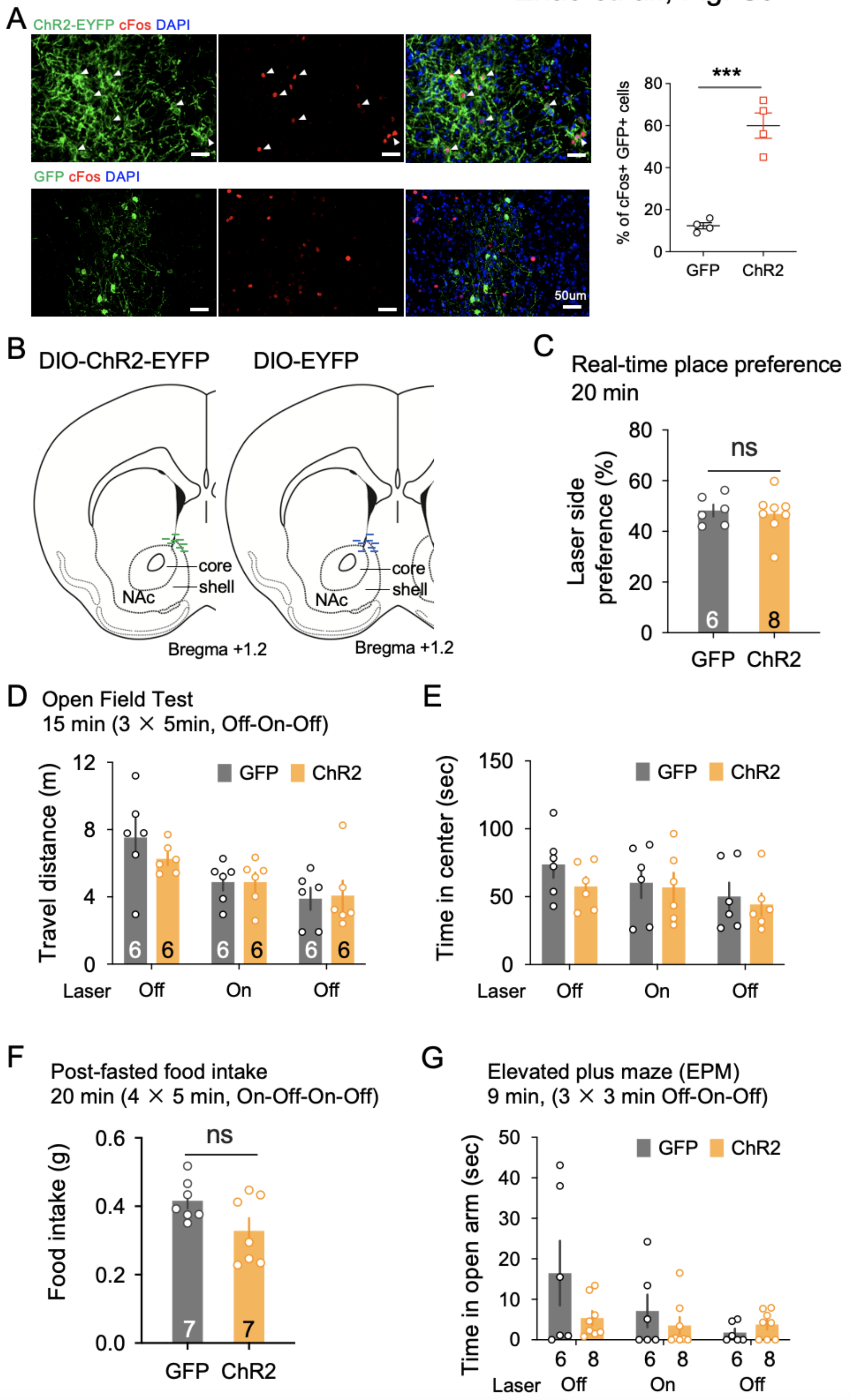

Fig. S3. Optogenetic activation of NAc *Tac2*$^+$ neurons

A. Laser stimulation (3 cycles of 3 min-on, 3 min-off) induces strong c-Fos expression in ChR2-EYFP expressing cells, but not in the control GFP-expressing cells. The ratio of cFos$^+$/GFP$^+$ cells in all GFP$^+$ cells was calculated and shown on the right panel. ***, p=0.0003, unpaired t-test.

B. Diagrams indicating the locations of implanted optic cannulas in the NAc of DIO-ChR2-EYFP expressing mice (green lines) and DIO-EYFP expressing mice (blue lines).

C-G. Optogenetic activation of the NAc *Tac2*-expressing neurons did not affect real-time place preference (**C**), locomotion in open field arena (**D**), time spent in the center area of open field arena (**E**), food intake (**F**), and elevated plus maze (**G**). Laser stimulation patterns are indicated in the figures. All p-values were calculated by unpaired t-test, ns, p>0.05.

Data in (A), (C), (D), (E), (F), and (G) are presented as mean ± SEM, scale bars of (A) are indicated in the figures.

Zhao et. al., Fig. S4

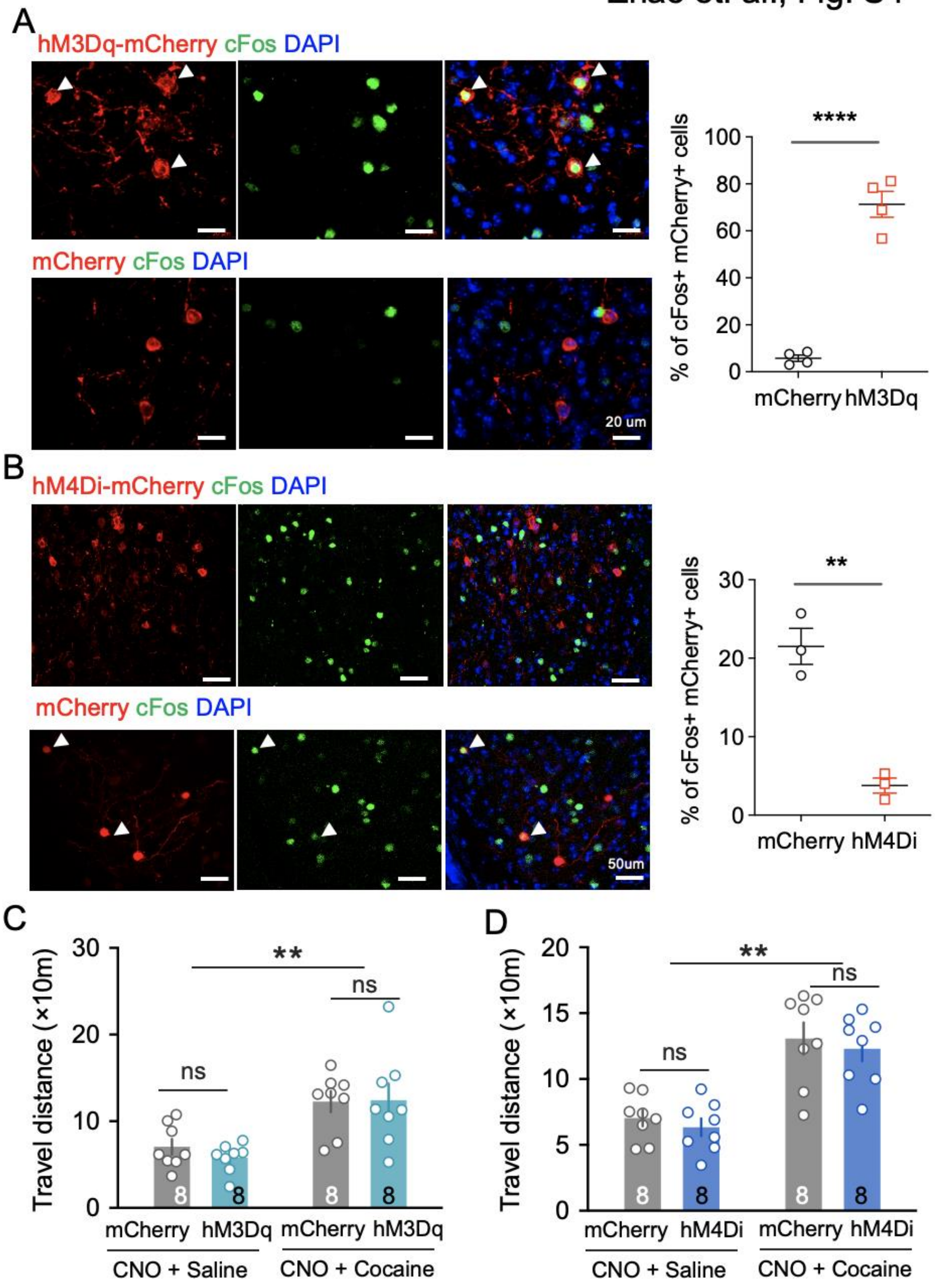

Fig. S4. Chemogenetic activation or inhibition of NAc *Tac2*⁺ neurons

A. cFos induction after intraperitoneal injection of ligand CNO in hM3Dq-mCherry-expressing and mCherry-expressing mice. The ratio of $cFos^+/mCherry^+$ cells in all $mCherry^+$ cells was calculated and shown on the right panel. ****, $p<0.0001$, unpaired t-test.

B. cFos induction after intraperitoneal injection of ligand CNO in hM4Di-mCherry-expressing and mCherry-expressing mice subjected to cocaine treatment. The ratio of $cFos^+/mCherry^+$ cells in all $mCherry^+$ cells was calculated and shown on the right panel. **, p=0.002, unpaired t-test.

C. Distance traveled in the 1-hour post-treatment period after chemogenetic excitation of *Tac2*$^+$ neurons. **, p≤0.01, ns, p>0.05, unpaired t-test.

D. Distance traveled in the 1-hour post-treatment period after chemogenetic inhibition of *Tac2*$^+$ neurons. **, p≤0.01, ns, p>0.05, unpaired t-test.

Data in (A), (B), (C), and (D) are presented as mean ± SEM, scale bars of (A) and (B) are indicated in the figures.

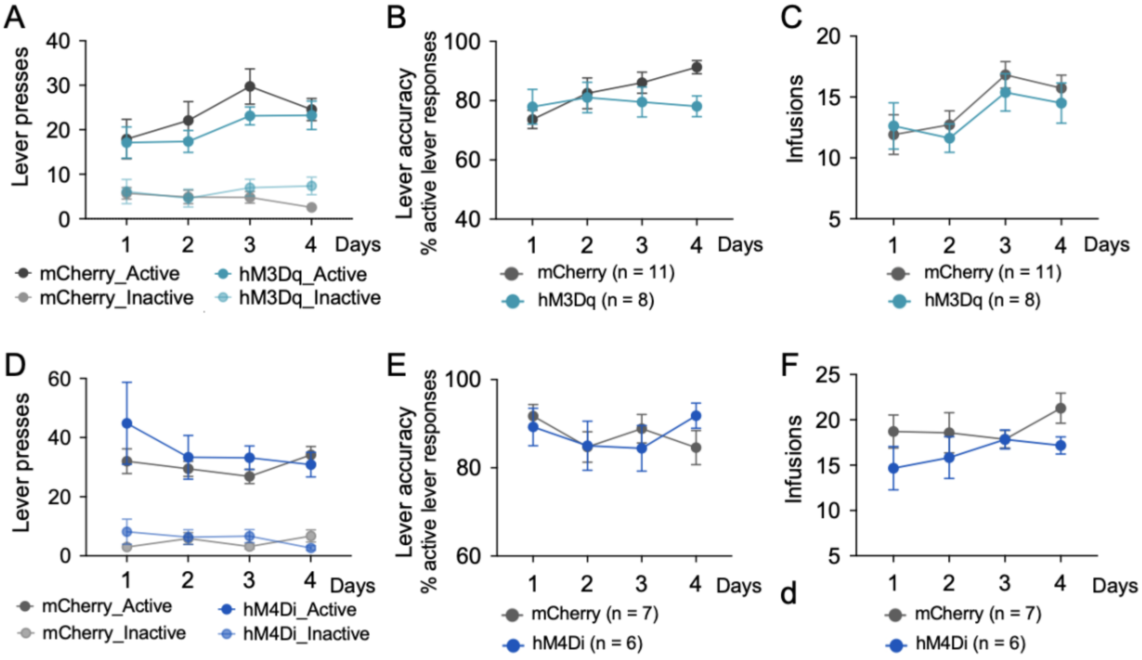

Fig. S5. Intravenous cocaine self-administration

A-B. The numbers of active lever press and inactive lever press (**A**), lever accuracy (**B**), and numbers of cocaine infusions (**C**) during the acquisition phase of cocaine IVSA training in response to chemogenetic activation of *Tac2*$^{+}$ neurons.

C-D. The numbers of active lever press and inactive lever press (**A**), lever accuracy (**B**), and numbers of cocaine infusions (**C**) during the acquisition phase of cocaine IVSA training in response to chemogenetic inhibition of *Tac2*$^{+}$ neurons.

Data in (A), (B), (C), (D), (E), and (F) are presented as mean ± SEM.

Zhao et. al., Fig. S6

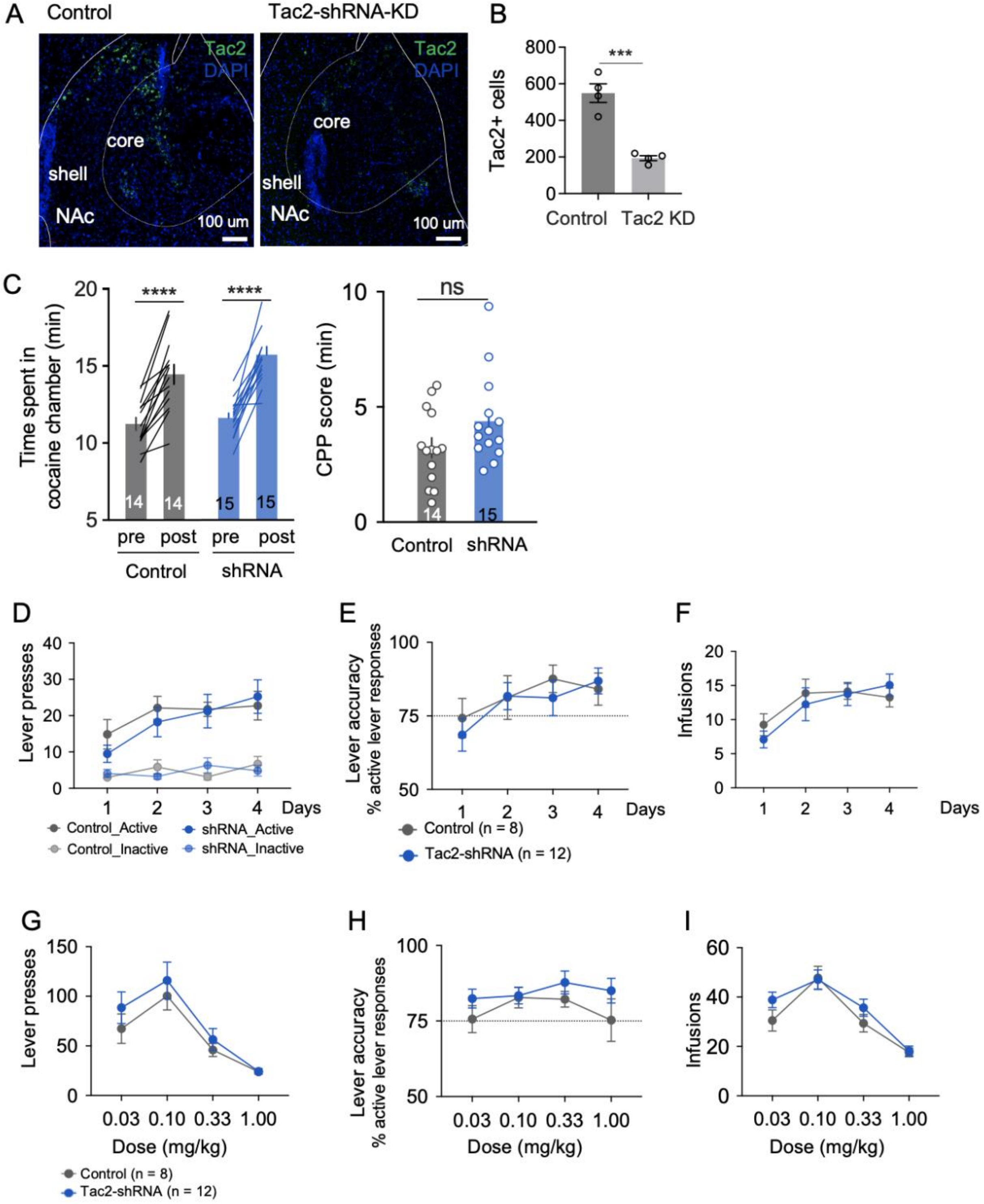

Fig. S6. **shRNA-mediated knock-down of Tac2 in NAc does not affect cocaine condition place preference nor contingent cocaine taking**

A. Representative *Tac2* RNA FISH images of mice injected with AAVs expressing Tac2 shRNA or control shRNA.

B. Quantification of *Tac2* knock-down efficiency. Numbers were calculated by summating *Tac2*$^{+}$ cells in serial 3 slides of the NAc region of individual mice (~Bregma +1.2).

C. Effects of *Tac2* KD on cocaine-CPP. Mice were subjected to 2-day conditioning, with each morning given saline injection followed by confined to saline-coupled chamber for 30 min, and in the afternoon, mice were given cocaine injection followed by confined to cocaine chamber for 30 min. Left panel: time spent in the cocaine-paired chambers pre- and post-conditioning, ****, $p<0.0001$, paired t-test. Right panel: the CPP scores were calculated by subtracting the time spent in pre-conditioning phase from the time spent in post-conditioning phase. ns, $p=0.0975$, unpaired t-test.

D-F. Cocaine-IVSA of Tac2 knock-down mice at acquisition phase. The numbers of active lever press and inactive lever press (**D**), lever accuracy (**E**), and numbers of cocaine infusions (**F**) during the acquisition phase of cocaine IVSA training.

G-I. Dose-dependent response under cocaine-IVSA of Tac2 knock-down mice. The numbers of lever presses (**G**), Lever accuracy (**H**), and numbers of infusions (**I**) are shown.

Data in (B), (C), (D), (E), (F), (G), (H), and (I) are presented as mean ± SEM. Scale bars of (A) are indicated in the figures.

Figure	Sample size (n)	Statistical test	P values
1E	saline: 6 mice<br>cocaine: 6 mice	two-tailed paired t test	saline (-10~0 vs 0~10 min): ns, p=0.3012;<br>cocaine (-10~0 vs 0~10 min): **, p=0.0046;<br>cocaine (0~10 vs 50~60 min): ns, p=0.1255
1G	saline: n=361 neurons<br>cocaine: n=356 neurons	two-sample Kolmogorov–Smirnov test	saline injection: -10~0min vs 0~10min, ns, p=0.9991;<br>cocaine injection: -10~0min vs 0~10min, **, p=0.0013;<br>-10~0min vs 50~60min, ns, p=,0.9715
1H	saline: n=361<br>cocaine: n=356	one-way ANOVA corrected with Benjamini and Yekutieli method	F (2, 1060) = 4.533, p=0.0110;<br>saline (0~10 min) vs cocaine (0~10 min): **, p=0.0043;<br>cocaine (0~10 min) vs cocaine (50~60 min): *, p=0.0250;<br>saline (0~10 min) vs cocaine (50~60 min): ns, p=0.7147
1I	excited neurons: n=49<br>inhibited neurons: n=117	one-way ANOVA corrected with Bonferroni's test	excited neurons: F (2, 144) = 57.07, ****, p<0.0001, **, p=0.0034;<br>inhibited neurons: F (2, 348) = 88.97 , ****, p<0.0001
2B	n=6 mice	two-tailed paired t test	**, p =0.0058
2D	n=6 mice	two-tailed paired t test	pre-conditioning: ns, p=0.5630<br>post-conditioning: **, p=0.0046
2E	excited neuron: n=16<br>inhibited neuron: n=62	two-tailed paired t-test	****p <0.0001
2F	n=16	two-tailed paired t-test	**, p =0.0059
2G	n=62	two-tailed paired t-test	****, p <0.0001
3C	GFP: n=8<br>ChR2: n=9	Left panel: two-tailed paired t-test<br>Right panel: two-tailed unpaired t-test	Left panel: ***, p=0.0007;<br>Right panel: *, p=0.0165
3D	GFP: n=6<br>ChR2: n=6	two-tailed unpaired t-test	****, p≤0.0001, ns, p>0.05
3F	mCherry: n=14<br>hM3Dq: n=13	Left panel: two-tailed paired t-test<br>Right panel: two-tailed unpaired t-test	Left panel: ***, p=0.0007, ns, p=0.560;<br>Right panel: **, p=0.007
3H	mCherry: n=7<br>hM3Dq: n=8	Left panel: two-tailed paired t-test<br>Right panel: two-tailed unpaired t-test	Left panel: ***, p=0.0009, ns, p=0.1027;<br>Right panel: *, p=0.0389
4B	mCherry: n=11<br>hM3Dq: n=8	Two-way ANOVA with Holm-Sidak's multiple comparisons test	dose: F (3, 68) = 2.916, p=0.0404;<br>virus: F (1, 68) = 15.22, p=0.0002;<br>interaction: F (3, 68) = 1.245, p=0.3002;<br>*, p=0.0455, 0.0344, and 0.0459, respectively; ns, p=0.7706
4C	mCherry: n=11<br>hM3Dq: n=8	two-tailed unpaired t test	**, p=0.010
4D	mCherry: n=11<br>hM3Dq: n=8	Two-way ANOVA with Holm-Sidak's multiple comparisons test	dose: F (3, 68) = 7.689, p=0.0002;<br>virus: F (1, 68) = 30.13, p<0.0001;<br>interaction: F (3, 68) = 1.723, p=0.1704;<br>**p=0.0069, ***p=0.0009; ns, p=0.3969
4E	mCherry: n=11<br>hM3Dq: n=8	Two-way ANOVA with Holm-Sidak's multiple comparisons test	virus, F (1, 102) = 13.87, p=0.0003;<br>time, F (5, 102) = 17.35, p<0.0001;<br>virus × time, F (5, 102) = 1.808, p=0.1178;<br>*, p=0.0416, **, p=0.0029.
4F	mCherry: n=7<br>hM4Di: n=6	Two-way ANOVA with Holm-Sidak's multiple comparisons test	virus: F (1, 44) = 13.88, p=0.0006;<br>dose: F (3, 44) = 14.07, p<0.0001;<br>interaction: F (3, 44) = 2.703, p=0.0569;<br>*, p=0381, **, p=0.0016.
4G	mCherry: n=7<br>hM4Di: n=6	two-tailed unpaired t test	ns, p=0.4939
4H	mCherry: n=7<br>hM4Di: n=6	Two-way ANOVA with Holm-Sidak's multiple comparisons test	dose: F (3, 44) = 6.772, p=0.0007;<br>virus: F (1, 44) = 9.708, p=0.0032;<br>interaction: F (3, 44) = 1.060, p=0.375;<br>*p=0.0357, ns, p>0.05
4I	mCherry: n=7<br>hM4Di: n=6	Two-way ANOVA with Holm-Sidak's multiple comparisons test	virus, F (1, 66) = 27.91, p<0.0001;<br>time, F (5, 66) = 31.88, p<0.0001;<br>virus × time, F (5, 66) = 0.5860, p=0.7106;<br>*, p=0.0474, **, p=0.0084

(*table continued on the next page*)

Figure	Sample size (n)	Statistical test	P values
5G	GFP: n=6<br>ChR2: n=5	two-tailed unpaired t-test	ns, p= 0.8795
5H	GFP: n=6<br>ChR2: n=9	two-tailed unpaired t-test	*,p=0.0181
5I	GFP: n=6<br>ChR2: n=6	two-tailed unpaired t-test	ns, p= 0.1544

Table. S1. Summary of statistical analyses.
